# The Hog as an Article of Food

**Published:** 1888-12

**Authors:** J. L. Cunningham

**Affiliations:** Dallas, Texas


### CORRESPONDENCE.

### THE HOG AS AN ARTICLE OF FOOD.

#### LETTER FROM DR. CUNNINGHAM.

*Editor Daniel's Texas Medical Journal:*

In the October number of your Journal, you made a vigorous protest against the use of hog's flesh in any form as an article of diet, and have exhausted the vocabulary of abuse in maligning the poor brute. I cannot agree with you:

1. That the product of the hog, flesh, lard or blood, is unhealthy.

2. That the hog is filthy in his habits.

The hog is omnivorous. He is a rustler. He will not starve if he can help it. He is "filthy" and unclean,"—a "scavenger" from necessity,—which knows no laws—and *not* from choice. He is more cleanly and tidy, than either the horse or the cow. He will not befoul his bed, as they do, if he can help it, but will keep everything neat and tidy[?]. He is choice as to what he eats; and will eat garbage only when he can do no better; and always prefers clean water to foul. I suppose, Doctor, you have no objection to the chicken, or the "product of the chicken" flesh, fat or eggs. Now what is the difference between a chicken and a pig, so far as "uncleanness," scavengering, etc., are concerned? Will the pig do any thing that the chicken will refuse to do? Will he go anywhere the latter will not follow? The duck is no less filthy then the chicken when forced to be so from necessity. If we reject the hog because of his filthy ways, to be consistent, we shall be compelled also to give up the chicken and the duck. What you say about the diseased product of the hogs—trichinous flesh, and lard made from "hogs dead of cholera,"—guts, hair, offal, etc., thrown in,—applies with equal force to the cow. She is frequently sick with charbon, pleuro-pneumonia, consumption, etc., and while affected, is sold for human food by unscrupulous men who, as butchers or milkmen, palm off as wholesome food, the flesh and milk of such diseased animals, on an unsuspecting public. It is not the *diseased* animal that we are considering, but the *healthy* one. Is the *healthy* hog a proper food for man, is the question we are considering. If you reject him because he sometimes gets sick, and while sick may be eaten as food. So must you reject, for like reasons, the cow. If you reject him because he is filthy, when necessity makes him so, to be consistent, you must likewise reject the chicken and the duck.

I am glad that you have inaugurated this crusade against the hog, inasmuch as the discussion of the subject will emphasize the great importance, aye, the urgent necessity for a rigid surveillance, by police authority, of our food products.

Now Doctor, notwithstanding my great penchant for spare-ribs and back-bone, sausage and blood pudding, at hog-killing time, and of boiled ham and breakfast bacon at all times, if you can demonstrate the fact that the hog,—the *healthy* hog—is an improper food product, I am willing to give up these luxuries, and join you in your crusade, and say to my old friend, "go." But until then, excuse me if you please. No one has a higher regard for Moses,—not even yourself,—as a sanitarian, than I have. In a paper which I read at the initial meeting of the Texas State Sanitary Association on the twentieth of October last, in the city of Dallas, I made the assertion, that with all our boasted learning and science, we were not able to improve upon the "Dry Earth" system, originated by Moses in dealing with human excreta. But on the subject of dietetics,—particularly as regards the hog as an article of human diet, I am not prepared to endorse him as an authority; the hog has stood the test of time and of the ages as a supreme artice of diet, and it will take more than ridicule and abuse, and *ipse dixits* to displace him. He has come to stay; and if I am not greatly mistaken *he will stay*.

Enclosed find $2 for subscription to the JOURNAL.

Respectfully, J. L. CUNNINGHAM.

Dallas, Texas, November 10, 1888.

